# Supplementary material for: Plasma Sphingomyelin Disturbances: Unveiling Its Dual Role as a Crucial Immunopathological Factor and a Severity Prognostic Biomarker in COVID-19
Source: Cells. 2023 Jul 26;12(15):1938. doi: 10.3390/cells12151938 (PMC10417089; doi:10.3390/cells12151938)
Supplement: Supplementary file 1 [file cells-12-01938-s001.zip › cells-2459439-supplementary.pdf]

cells

MDPI

Supplementary Material

Plasma Sphingomyelin Disturbances: Unveiling Its Dual Role as a Crucial Immunopathological Factor and a Severity Prognostic Biomarker in COVID-19

Diana Mota Toro [1,2,†], Pedro V. da Silva-Neto [1,2,†], Jonatan C. S. de Carvalho [1,3,†], Carlos A. Fuzo [1,†], Malena M. Pérez [1], Vinícius E. Pimentel [1,4], Thais F. C. Fraga-Silva [4], Camilla N. S. Oliveira [1,4], Glaucia R. Caruso [1], Adriana F. L. Vilela [3], Pedro Nobre-Azevedo [3,4], Thiago V. Defelippo-Felippe [3], Jamille G. M. Argolo [5], Augusto M. Degiovani [6], Fátima M. Ostini [6], Marley R. Feitosa [7,8], Rogerio S. Parra [7,8], Fernando C. Vilar [8,9], Gilberto G. Gaspar [9], José J. R. da Rocha [7], Omar Feres [7,8], Gabriel P. Costa [10], Sandra R. C. Maruyama [11], Elisa M. S. Russo [1], Ana Paula M. Fernandes [5], Isabel K. F. M. Santos [4], Adriana Malheiro [2], Ruxana T. Sadikot [12], Vânia L. D. Bonato [4], Cristina R. B. Cardoso [1], Marcelo Dias-Baruffi [1], Átila A. Trapé [10,‡], Lúcia H. Faccioli [1,‡] and Carlos A. Sorgi [2,3,4,‡,*] and ImmunoCovid Consortium Group [§]

*** Correspondence:** Carlos A. Sorgi **-** carlos.sorgi@usp.br +55 16 3315-9176
[†] These authors contributed equally to this work and share first authorship
[#] These authors contributed equally to this work and share senior authorship

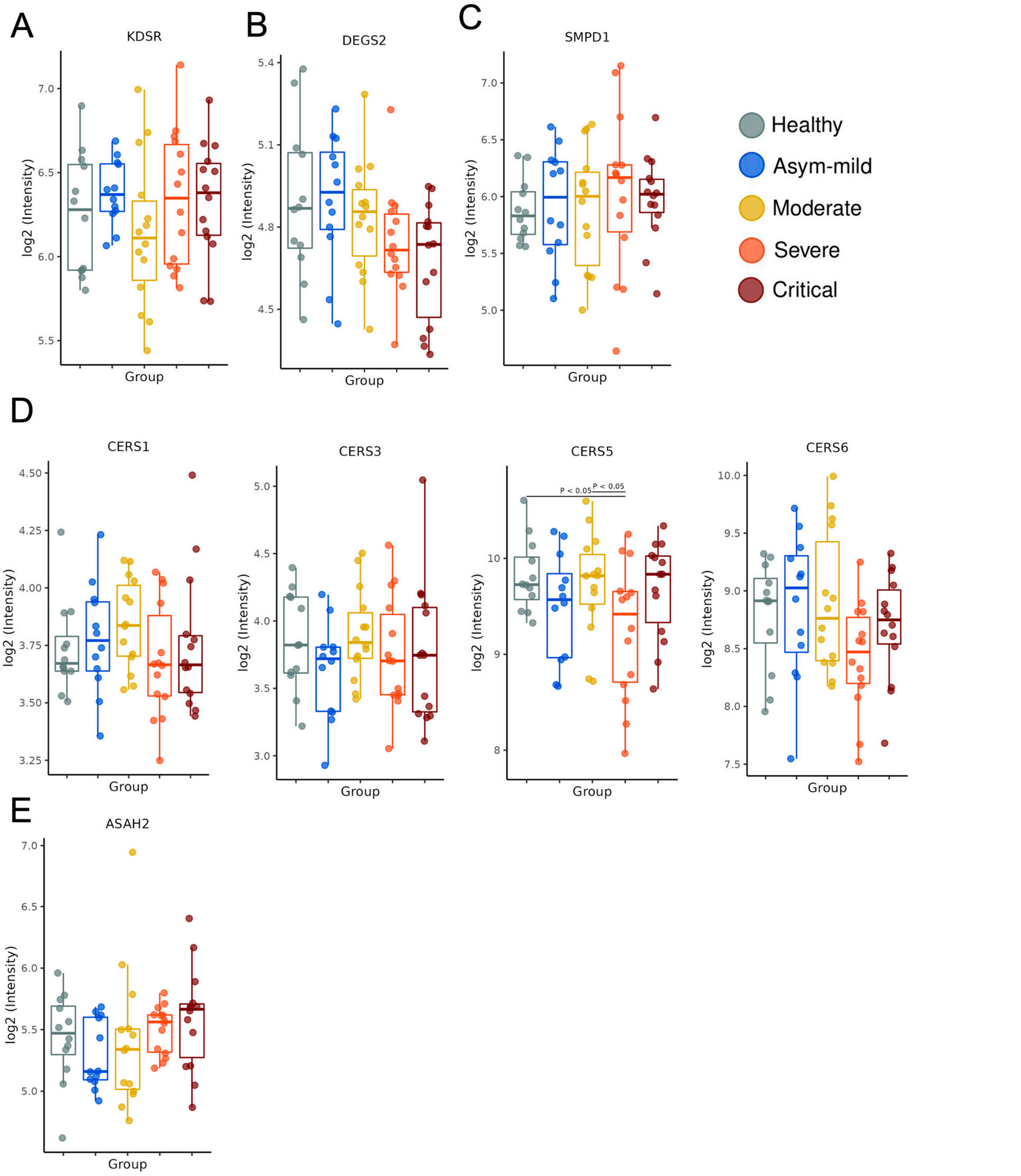

Supplementary Figure S1. Expression of sphingolipid metabolic enzymes in patients with COVID-19 according to severity. (**A**) 3-ketodihydrosphingosine reductase (*KDSR*), (**B**) Sphingolipid delta (4)-desaturase DES2 (*DEGS2*), (**C**) Sphingomyelin phosphodiesterase (*SMPD* 1), (**D**) Ceramide synthase 1, 3, 4, 5 (*CERS1*/3/5/6), (**E**) N-acylsphingosine amidohydrolase 2 (ASAH2), in: Control (n=12), Asy-to-mild (n=12), moderate (n=14), severe (n=14) and critical (n=14). The log2 of normalized gene expression profiles for analyzed groups at are showed as boxplots. Significant differences in transcript expression correspond to Benjamini and Hockberg adjusted p-values obtained from whole transcriptome differential expression analysis considering a threshold of < 0.05 in at least one pair of clinical groups. Details of enzyme nomenclatures Supplementary Table 4.

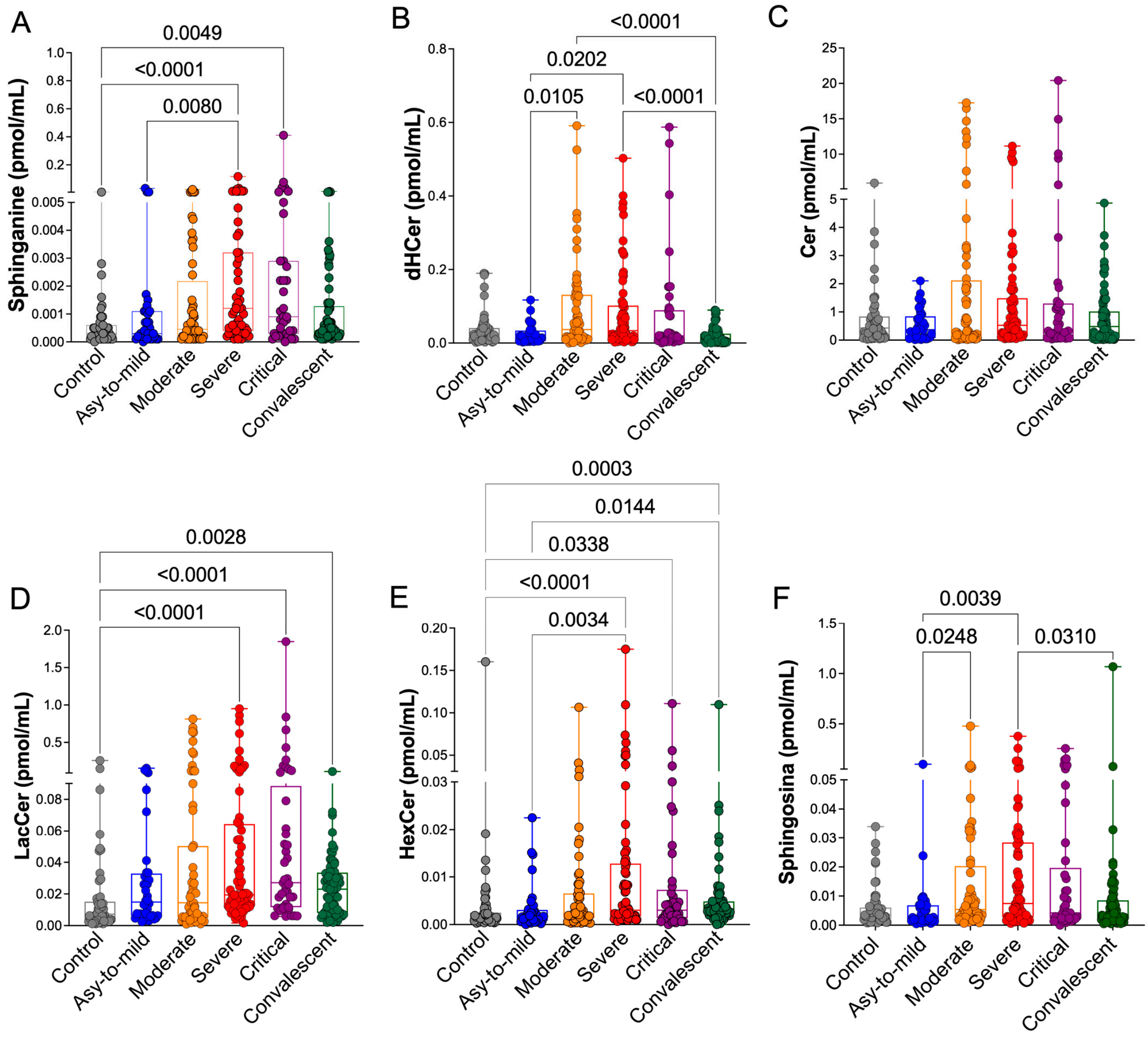

Supplementary Figure S2. Sphingolipids abundance profile in patients with COVID-19. LC–MS/MS measurements for (**A**) Sphinganine (18:0), (**B**) dihydroceramide (dHCer), (**C**) Ceramide (Cer), (**D**) Lactosylceramide 16:0 (LacCer), (**E**) Hexaglycosylceramide (HexCer) and (**F**) Sphingosine. Statistical analyzes were performed using the Kruskal-Wallis multiple comparison test (non-parametric), followed by the Dunn post-test for pairwise comparison. Data are expressed as median in boxplot graphics. Significance levels shown are based on statistically significant between groups with *p*-value < 0.05.

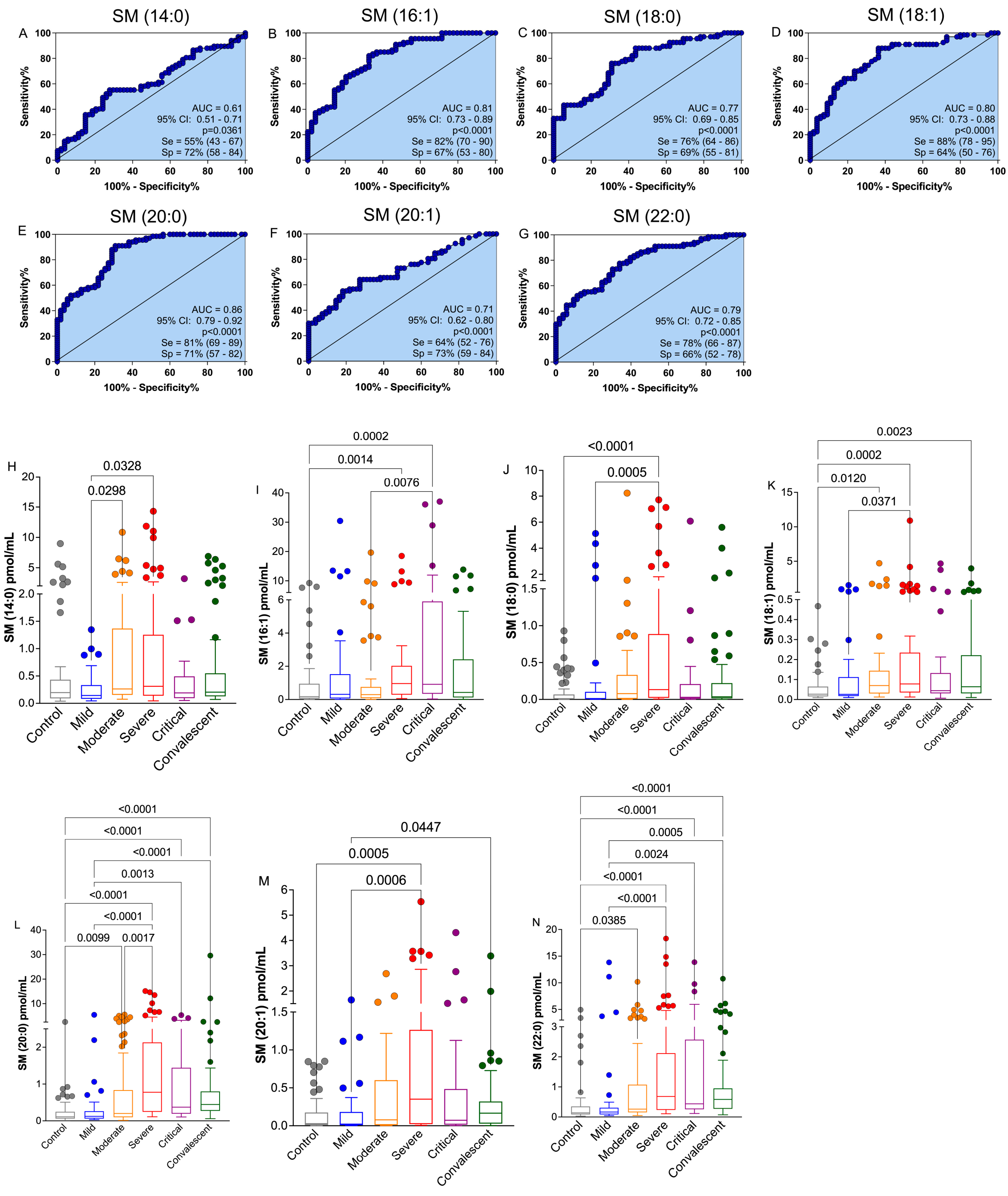

Supplementary Figure S3. ROC curves and Analysis of serum abundance sphingomyelin (SM) species (14:0), (16:1), (18:0), (18:1), (20:0), (20:1) and (22:0) in COVID-19. (**A-G**) ROC curves for sphingomyelin species (SM) in patients with severe forms of Covid-19. The curves compare the severe patients groups (n= 67) with the control (n=55). AUC: area under the curve; Se: sensitivity; Sp: specificity; CI: 95% confidence interval. (**H-N**) sphingomyelin class profile in control subjects compared to patients with COVID-19 and convalescents in: mild (n=36), moderate (n=60), severe (n=67), critical (n=41) and Convalescent individuals (n=77). Statistical analyzes were performed using the Kruskal-Wallis multiple comparison test (non-parametric), followed by the Dunn post-test for pairwise comparison. Data are expressed as median in boxplot graphics with minimum and maximum values. Significance levels shown are based on statistically significant between groups with *p*-value < 0.05.

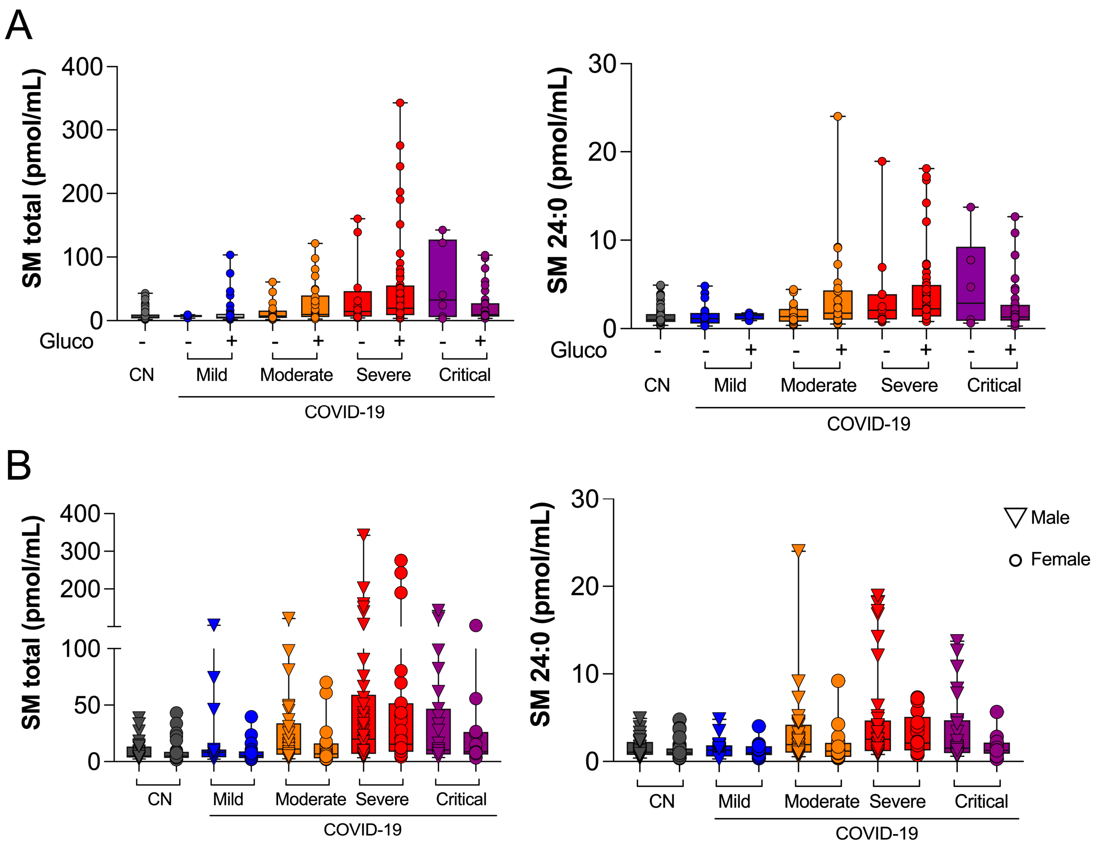

Supplementary Figure S4. Glucocorticoid therapy and sex differences have no impact on total SM and (24:0)-specie production. Analysis of total sphingomyelin and SM (24:0) levels in relation to the use of (**A**) glucocorticoids and (**B**) male and female sex. Statistical analyzes were performed using the Kruskal-Wallis multiple comparison test (non-parametric), followed by the Dunn post-test for pairwise comparison. Data are expressed as median in boxplot graphics. Significance levels shown are based on statistically significant between groups with p-value < 0.05.

$$(I)\ AUC_{n(k)total} = AUC_{n(k)}\ (1 + 0.0109n + \frac{0.0109^2 n(n-1)}{2}$$

$$(II)\ C_{lipid} = \frac{AUC_{lipid}}{AUC_{IS}} * C_{IS}$$

Supplementary Figure S5. Lipid quantification using internal standard and monoisotopic correction. (I) Monoisotopic correction: $AUC_{n(k)}$ total = corrected analyte area under the curve, $AUC_{n(k)}$ = quantified analyte monoisotopic mass area under the curve, n = number of C atoms, k = number of double bonds, **(II)** Lipid quantification based on the internal standard: C_{lipid} = analyte concentration (pmol/mL), AUC_{lipid} = monoisotopically corrected analyte area under the curve by Equation I ($AUC_{n(k)total}$), AUC_{IS} = area under the curve of internal standard corresponding to the analyte family, C_{IS} = internal standard concentration according to the Certificate of Analysis (Lot Number: LM6004-LM40-112A, Avanti Polar Lipids) [29].

Supplementary Table S1: Sphingolipid subclasses and species detected in our cohort.

Subclasses	Number of species	Species Description
**Ceramide**	9	Cer (16:0), Cer (18:0), Cer (18:1), Cer (20:0), Cer (22:0), Cer (23:0), Cer (24:0), Cer (24:1), Cer (25:0)
**Sphingosine**	2	Sphingosine (14:0), Sphingosine (18:0)
**Sphinganine**	1	Sphinganine (18:0)
**Sphingomyelin (SM)**	17	SM (14:0), SM (16:0), SM (16:1), SM (18:0), SM (18:1), SM (20:0), SM (20:1), SM (22:0), SM (22:1), SM (23:0), SM (23:1), SM (24:0), SM (24:1), SM (25:0), SM (25:1), SM (26:0), SM (26:1).
**Hexaglycosylceramide (HexCer)**	2	HexCer (16:0), HexCer (24:0)
**Lactosylceramide (LacCer)**	2	LacCer (16:0), LacCER(d18:1/14:0)
**Dihydroceramide (dHCer)**	5	dHCer(d18:0/16:0), dHCer(d18:0/18:0), dHCer(d18:0/24:0), dHCer(d20:0/16:0), dHCer(d20:0/22:0)
Total	38	

Supplementary Table S2. Target high-resolution mass spectrometry parameters for SL species.

Experiment	Precursor ion (m/z)	Analyte	Internal Standard	Fragment ion (m/z)
1	100-2000	TOF-MS (untargeted)	-	-
2	*552,5*	Ceramide (d18:1/17:0)	*IS*	*534.5237-534.5297*
3	*647,5*	SM (d18:1/12:0)	*IS Mix II (Avanti)*	*184.0703-184.0753*
4	*286,2*	Sphingosine C17:0	*IS Mix II (Avanti)*	*268.2623-268.2663*
5	*288,2*	Sphinganine C17:0	*IS Mix II (Avanti)*	*270.2750-270.2810*
6	*368,2*	Sphinganine-1-P C17:0	*IS Mix II (Avanti)*	*270.2750-270.2830*
7	*482,4*	Ceramide (d18:1/12:0)	*IS Mix II (Avanti)*	*264.2653-264.2703*
8	*644,5*	GlcCer (d18:1/12:0)	*IS Mix II (Avanti)*	*264.2652-264.26702*
9	*806,5*	LacCer (d18:1/12:0)	*IS Mix II (Avanti)*	*264.2652-264.26702*
10	*538,5*	Ceramide (d18:1/16:0)	*Ceramide (d18:1/12:0)*	*264.2652 - 264.26710*
11	*566,5*	Ceramide (d18:1/18:0)	*Ceramide (d18:1/12:0)*	*264.2652 - 264.26704*
12	*648,6*	Ceramide (d18:1/24:1)	*Ceramide (d18:1/12:0)*	*264.2652 - 264.26802*
13	*650,6*	Ceramide (d18:1/24:0)	*Ceramide (d18:1/12:0)*	*264.2652 - 264.26702*
14	*664,4*	Ceramide (d18:1/25:0)	*Ceramide (d18:1/12:0)*	*264.2652 - 264.26702*
15	*700,5*	GlcCer (d18:1/16:0)	*GlcCer (d18:1/12:0)*	*264.2652 - 264.26702*
16	*728,6*	GlcCer (d18:1/18:0)	*GlcCer (d18:1/12:0)*	*264.2652 - 264.26702*
17	*810,6*	GlcCer (d18:1/24:1)	*GlcCer (d18:1/12:0)*	*264.2652 - 264.26702*
18	*731,6*	SM (d18:1/18:0)	*SM (d18:1/12:0)*	*184.0703 - 184.0753*
19	*815,7*	SM (d18:1/24:0)	*SM (d18:1/12:0)*	*184.0703 - 184.0753*
20	*862,6*	LacCer (d18:1/16:0)	*LacCer (d18:1/12:0)*	*264.2652 - 264.26702*
21	*974,7*	LacCer (d18:124:0)	*LacCer (d18:1/12:0)*	*264.2652 - 264.26702*
22	*300,3*	Sphingosine C18:0	*Sphingosine C17:0*	*282.2480 - 282.2530*
23	*272,2*	Sphingosine C16:0	*Sphingosine C17:0*	*254.2164 - 254.2214*
24	*244,2*	Sphingosine C14:0	*Sphingosine C17:0*	*226.1765 - 226.1815*
25	*302,2*	Sphinganine C18:0	*Sphinganine C17:0*	*284.2912 - 284.2952*
26	*274,2*	Sphinganine C16:0	*Sphinganine C17:0*	*256.2612 - 256.2652*
27	*382,2*	Sphinganine-1-P C18:0	*Sphinganine-1-P C17:0*	*364.2120 - 364.2160*
28	354,2	Sphinganine-1-P C16:0	*Sphinganine-1-P C17:0*	*336.2298 - 336.2338*
29	*356,35*	Sphingosine C22:1	*Sphingosine C17:0*	*338.3500 - 338.3540*
30	*328,3*	Sphingosine C20:1	*Sphingosine C17:0*	*310.3000 - 310.3040*
31	*330,3*	Sphinganine C20:0	*Sphinganine C17:0*	*214.3000 - 214.3040*
32	*410,3*	Sphinganine-1-P C20:0	*Sphinganine C17:0*	*392.3000 - 392.3040*
33	*464,4*	Ceramide (d18:1/18:1)	*Ceramide (d18:1/12:0)*	*264.2694 - 264.2714*
34	*594,6*	Ceramide (d18:1/20:0)	*Ceramide (d18:1/12:0)*	*264.2671 - 264.2701*
35	*622,7*	Ceramide (d18:1/22:0)	*Ceramide (d18:1/12:0)*	*264.2671 - 264.2701*

36	*634,7*	Ceramide (d18:1/23:1)	*Ceramide (d18:1/12:0)*	*264.2671 - 264.2701*
37	*636,7*	Ceramide (d18:1/23:0)	*Ceramide (d18:1/12:0)*	*264.2671 - 264.2701*
38	*662,8*	Ceramide (d18:1/25:1)	*Ceramide (d18:1/12:0)*	*264.2671 - 264.2701*
39	*676,8*	Ceramide (d18:1/26:1)	*Ceramide (d18:1/12:0)*	*264.2671 - 264.2701*
40	*678,8*	Ceramide (d18:1/26:0)	*Ceramide (d18:1/12:0)*	*264.2671 - 264.2701*
41	*675,5*	SM (d18:1/14:0)	*SM (d18:1/12:0)*	*183.9758 - 184.0758*
42	*701,6*	SM (d18:1/16:0)	*SM (d18:1/12:0)*	*183.9758 - 184.0758*
43	*703,57*	SM (d18:1/16:0)	*SM (d18:1/12:0)*	*183.9758 - 184.0758*
44	*729,6*	SM (d18:1/18:1)	*SM (d18:1/12:0)*	*183.9758 - 184.0758*
45	*757,6*	SM (d18:1/20:1)	*SM (d18:1/12:0)*	*183.9758 - 184.0758*
46	*759,6*	SM (d18:1/20:0)	*SM (d18:1/12:0)*	*183.9758 - 184.0758*
47	*785,591*	SM (d18:1/22:1)	*SM (d18:1/12:0)*	*183.9758 - 184.0758*
48	*787,6*	SM (d18:1/22:0)	*SM (d18:1/12:0)*	*183.9758 - 184.0758*
49	*799,6*	SM (d18:1/23:1)	*SM (d18:1/12:0)*	*183.9758 - 184.0758*
50	*801,6*	SM (d18:1/23:0)	*SM (d18:1/12:0)*	*183.9758 - 184.0758*
51	*813,7*	SM (d18:1/24:1)	*SM (d18:1/12:0)*	*183.9758 - 184.0758*
52	*827,7*	SM (d18:1/25:1)	*SM (d18:1/12:0)*	*183.9758 - 184.0758*
53	*829,7*	SM (d18:1/25:0)	*SM (d18:1/12:0)*	*183.9758 - 184.0758*
54	*841,7*	SM (d18:1/26:1)	*SM (d18:1/12:0)*	*183.9758 - 184.0758*
55	*843,7*	SM (d18:1/26:0)	*SM (d18:1/12:0)*	*183.9758 - 184.0758*
56	*726,58*	HexCer (d18:1/18:1)	*GlcCer (d18:1/12:0)*	*264.2652 - 264.26702*
57	*754,7*	HexCer (d18:1/20:1)	*GlcCer (d18:1/12:0)*	*264.2652 - 264.26703*
58	*756,7*	HexCer (d18:1/20:0)	*GlcCer (d18:1/12:0)*	*264.2652 - 264.26704*
59	*782,7*	HexCer (d18:1/22:1)	*GlcCer (d18:1/12:0)*	*264.2652 - 264.26705*
60	*784,66*	HexCer (d18:1/22:0)	*GlcCer (d18:1/12:0)*	*264.2652 - 264.26706*
61	*796,7*	HexCer (d18:1/23:1)	*GlcCer (d18:1/12:0)*	*264.2652 - 264.26707*
62	*798,7*	HexCer (d18:1/23:0)	*GlcCer (d18:1/12:0)*	*264.2652 - 264.26708*
63	*808,6*	HexCer (d18:1/24:2)	*GlcCer (d18:1/12:0)*	*264.2652 - 264.26709*
64	*812,7*	HexCer (d18:1/24:0)	*GlcCer (d18:1/12:0)*	*264.2652 - 264.26710*
65	*824,6*	HexCer (d18:1/25:1)	*GlcCer (d18:1/12:0)*	*264.2652 - 264.26711*
66	*826,8*	HexCer (d18:1/25:0)	*GlcCer (d18:1/12:0)*	*264.2652 - 264.26712*
67	*836,6*	HexCer (d18:1/26:2)	*GlcCer (d18:1/12:0)*	*264.2652 - 264.26713*
68	*838,8*	HexCer (d18:1/26:1)	*GlcCer (d18:1/12:0)*	*264.2652 - 264.26714*
69	*840,8*	HexCer (d18:1/26:0)	*GlcCer (d18:1/12:0)*	*264.2652 - 264.26715*
70	*716,7*	HexCer OH (d18:1/16:0)	*GlcCer (d18:1/12:0)*	*264.2652 - 264.26716*
71	*744,7*	HexCer OH (d18:1/18:0)	*GlcCer (d18:1/12:0)*	*264.2652 - 264.26717*

72	*772,7*	HexCer OH (d18:1/20:0)	*GlcCer (d18:1/12:0)*	*264.2652 - 264.26718*
73	*798,7*	HexCer OH (d18:1/22:1)	*GlcCer (d18:1/12:0)*	*264.2652 - 264.26719*
74	*800,7*	HexCer OH (d18:1/22:0)	*GlcCer (d18:1/12:0)*	*264.2652 - 264.26720*
75	*812,7*	HexCer OH (d18:1/23:1)	*GlcCer (d18:1/12:0)*	*264.2652 - 264.26721*
76	*814,7*	HexCer OH (d18:1/23:0)	*GlcCer (d18:1/12:0)*	*264.2652 - 264.26722*
77	*826,8*	HexCer OH (d18:1/24:1)	*GlcCer (d18:1/12:0)*	*264.2652 - 264.26723*
78	*828,8*	HexCer OH (d18:1/24:0)	*GlcCer (d18:1/12:0)*	*264.2652 - 264.26724*
79	*840,8*	HexCer OH (d18:1/25:1)	*GlcCer (d18:1/12:0)*	*264.2652 - 264.26725*
80	*842,8*	HexCer OH (d18:1/25:0)	*GlcCer (d18:1/12:0)*	*264.2652 - 264.26726*
81	*854,8*	HexCer OH (d18:1/26:1)	*GlcCer (d18:1/12:0)*	*264.2652 - 264.26727*
82	*856,8*	HexCer OH (d18:1/26:0)	*GlcCer (d18:1/12:0)*	*264.2652 - 264.26728*
83	*974,7502*	LacCer (d18:0/24:1)	*LacCer (d18:1/12:0)*	*264.2687 - 264.2727*
84	*972,7346*	LacCer (d18:1/24:1(15Z)	*LacCer (d18:1/12:0)*	*264.2687 - 264.2728*
85	*1002,7815*	LacCer (d18:0/26:1)	*LacCer (d18:1/12:0)*	*264.2687 - 264.2729*
86	*1000,7659*	LacCer (d18:1/26:1(17Z))	*LacCer (d18:1/12:0)*	*264.2687 - 264.2730*
87	*886,6407*	LacCer (d18:1/18:1(9Z))	*LacCer (d18:1/12:0)*	*264.2687 - 264.2731*
88	*889,6563*	LacCer (d18:0/18:1)	*LacCer (d18:1/12:0)*	*264.2687 - 264.2732*
89	*948,7346*	LacCer (d18:0/22:0)	*LacCer (d18:1/12:0)*	*264.2687 - 264.2733*
90	*946,7189*	LacCer (d18:1/22:0)	*LacCer (d18:1/12:0)*	*264.2687 - 264.2734*
91	*920,7033*	LacCer (d18:0/20:0)	*LacCer (d18:1/12:0)*	*264.2687 - 264.2735*
92	*918,6876*	LacCer (d18:1/20:0)	*LacCer (d18:1/12:0)*	*264.2687 - 264.2736*
93	*1004,7972*	LacCer (d18:0/26:0)	*LacCer (d18:1/12:0)*	*264.2687 - 264.2737*
94	*1002,7815*	LacCer(d18:1/26:0)	*LacCer (d18:1/12:0)*	*264.2687 - 264.2738*
95	*864,6407*	LacCer (d18:0/16:0)	*LacCer (d18:1/12:0)*	*264.2687 - 264.2739*
96	*806,5624*	LacCer (d14:1/16:0)	*LacCer (d18:1/12:0)*	*264.2687 - 264.2740*
97	*892,672*	LacCer (d18:0/18:0)	*LacCer (d18:1/12:0)*	*264.2687 - 264.2741*
98	*836,6094*	LacCer (d14:0/18:0)	*LacCer (d18:1/12:0)*	*264.2687 - 264.2742*
99	*890,6563*	LacCer (d18:1/18:0)	*LacCer (d18:1/12:0)*	*264.2687 - 264.2743*
100	*976,7659*	LacCer (d18:0/24:0)	*LacCer (d18:1/12:0)*	*264.2687 - 264.2744*
101	*974,7502*	LacCer (d18:1/24:0)	*LacCer (d18:1/12:0)*	*264.2687 - 264.2745*
102	*836,6094*	LacCer (d18:0/14:0)	*LacCer (d18:1/12:0)*	*264.2687 - 264.2746*
103	*834,5937*	LacCer (d18:1/14:0)	*LacCer (d18:1/12:0)*	*264.2687 -264.2747*
104	*484,465*	dHCer (d18:0/12:0)	*Ceramide (d18:1/12:0)*	*264.2687 -264.2747*
105	*540,535*	dHCer (d18:0/16:0)	*Ceramide (d18:1/12:0)*	*284.2943 - 284.2983*
106	*568,563*	dHCer (d18:0/18:0)	*Ceramide (d18:1/12:0)*	*284.2943 - 284.2983*
107	*596,5976*	dHCer (d18:0/20:0)	*Ceramide (d18:1/12:0)*	*284.2943 - 284.2983*
108	*624,6289*	dHCer (d18:0/22:0)	*Ceramide (d18:1/12:0)*	*284.2943 - 284.2983*

109	*650,6446*	dHCer (d18:0/24:1(15Z))	*Ceramide (d18:1/12:0)*	*284.2943 - 284.2983*
110	*652,6602*	dHCer (d18:0/24:0)	*Ceramide (d18:1/12:0)*	*284.2943 - 284.2983*
111	*678,6759*	dHCer (d18:0/26:1(17Z))	*Ceramide (d18:1/12:0)*	*284.2943 - 284.2983*
112	*680,6915*	dHCer (d18:0/26:0)	*Ceramide (d18:1/12:0)*	*284.2943 - 284.2983*
113	*566,5507*	dHCer (d18:0/18:1)	*Ceramide (d18:1/12:0)*	*284.2943 - 284.2983*
114	*512,5037*	dHCer (d18:0/14:0)	*Ceramide (d18:1/12:0)*	*284.2943 - 284.2983*
115	*668,6551*	dHCer (d18:0/h24:0)	*Ceramide (d18:1/12:0)*	*284.2943 - 284.2983*
116	*696,6864*	dHCer (d18:0/h26:0)	*Ceramide (d18:1/12:0)*	*284.2943 - 284.2983*
117	*570,5456*	dHCer (d18:0/h17:0)	*Ceramide (d18:1/12:0)*	*284.2943 - 284.2983*
118	*498,4881*	dHCer (d18:0/13:0)	*Ceramide (d18:1/12:0)*	*284.2943 - 284.2983*
119	*526,5194*	dHCer (d18:0/15:0)	*Ceramide (d18:1/12:0)*	*284.2943 - 284.2983*
120	*554,5507*	dHCer (d18:0/17:0)	*Ceramide (d18:1/12:0)*	*284.2943 - 284.2983*
121	*568,3566*	dHCer (d20:0/16:0)	*Ceramide (d18:1/12:0)*	*284.2943 - 284.2983*
122	*596,5976*	dHCer (d20:0/18:0)	*Ceramide (d18:1/12:0)*	*284.2943 - 284.2983*
123	*624,6289*	dHCer (d20:0/20:0)	*Ceramide (d18:1/12:0)*	*284.2943 - 284.2984*
124	*652,6602*	dHCer (d20:0/22:0)	*Ceramide (d18:1/12:0)*	*284.2943 - 284.2985*
125	*680,6915*	dHCer (d20:0/24:0)	*Ceramide (d18:1/12:0)*	*284.2943 - 284.2986*
126	*708,7228*	dHCer (d20:0/26:0)	*Ceramide (d18:1/12:0)*	*284.2943 - 284.2987*

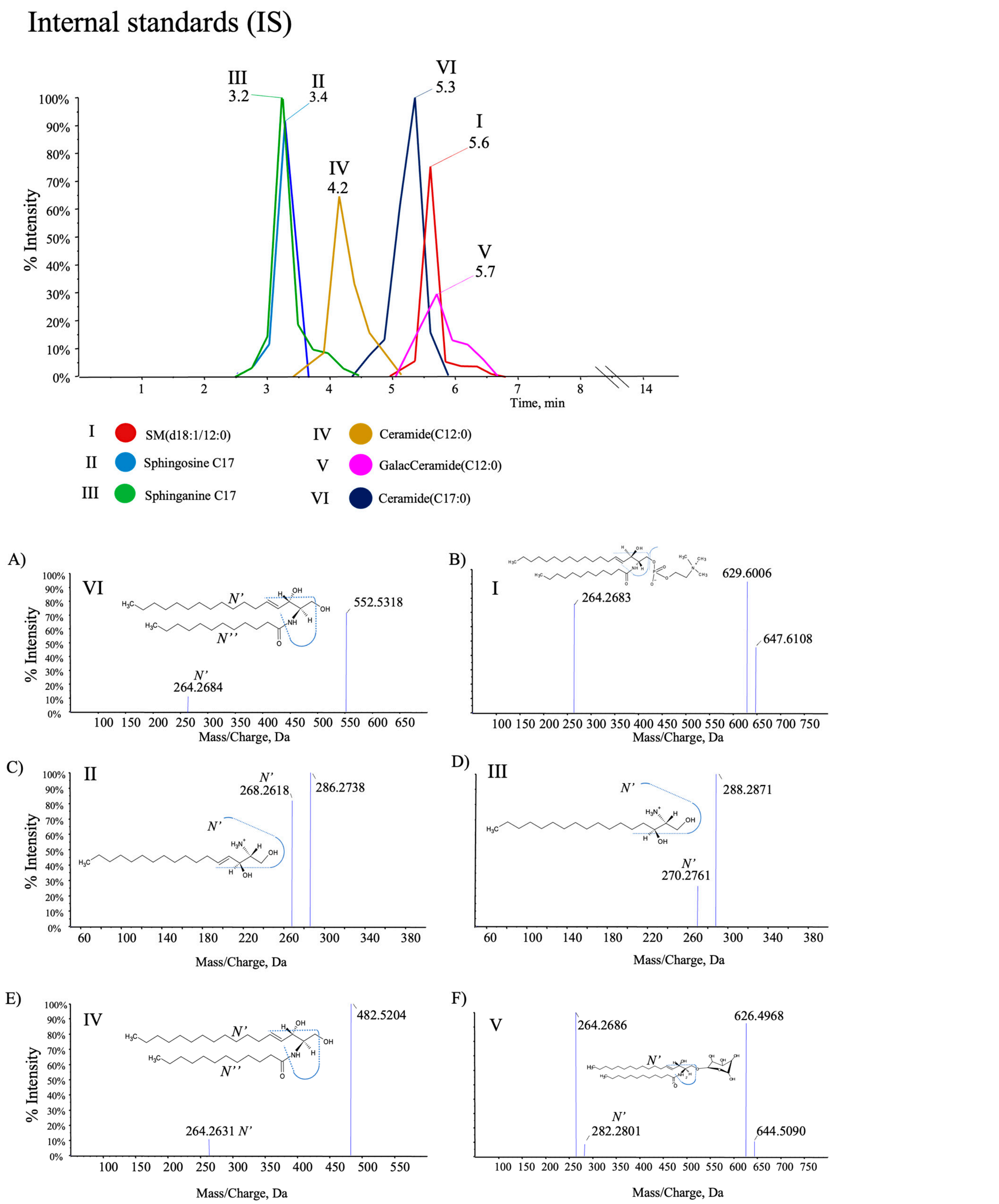

Supplementary Figure S6. Chromatographic profile and mass spectral data of internal standards used as reference for analysis. (I) Sphingomyelin (d18:0/12:0). (II) Sphingosine (17:0). (III) Sphinganine (17:0). (IV) Ceramide (12:0). (V) Galactosylceramide (12:0). (VI) Ceramide (17:0).

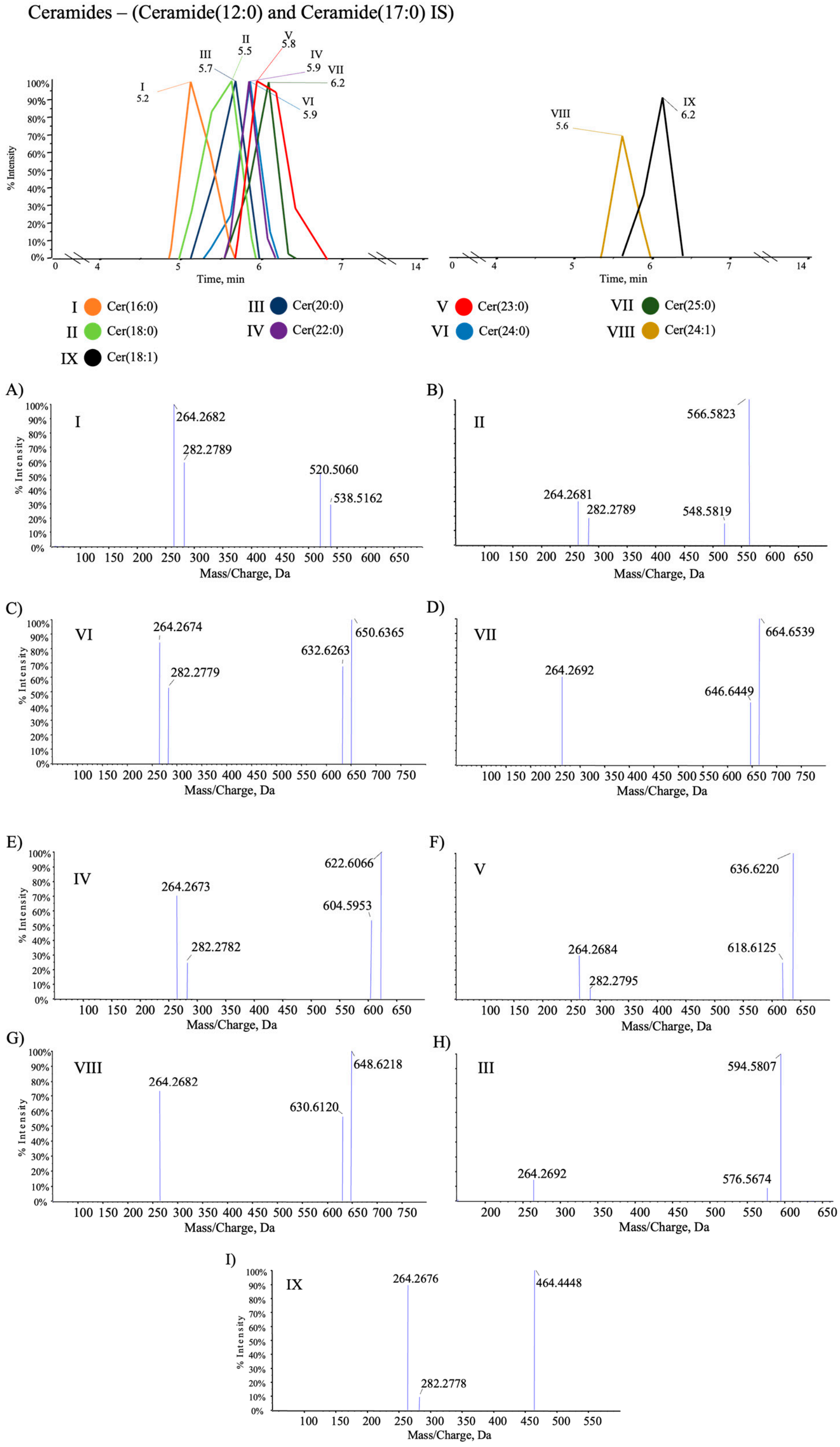

Supplementary Figure S7. Chromatographic profile and mass spectral data of detected ceramides. (I) Ceramide (16:0). (II) Ceramide (18:0). (III) Ceramide (20:0). (IV) Ceramide (22:0). (V) Ceramide (23:0). (VI) Ceramide (24:0). (VII) Ceramide (25:0). (VIII) Ceramide (24:1). (IX) Ceramide (18:1).

Sphingomyelin – (SM(d18:1/12:0) IS)

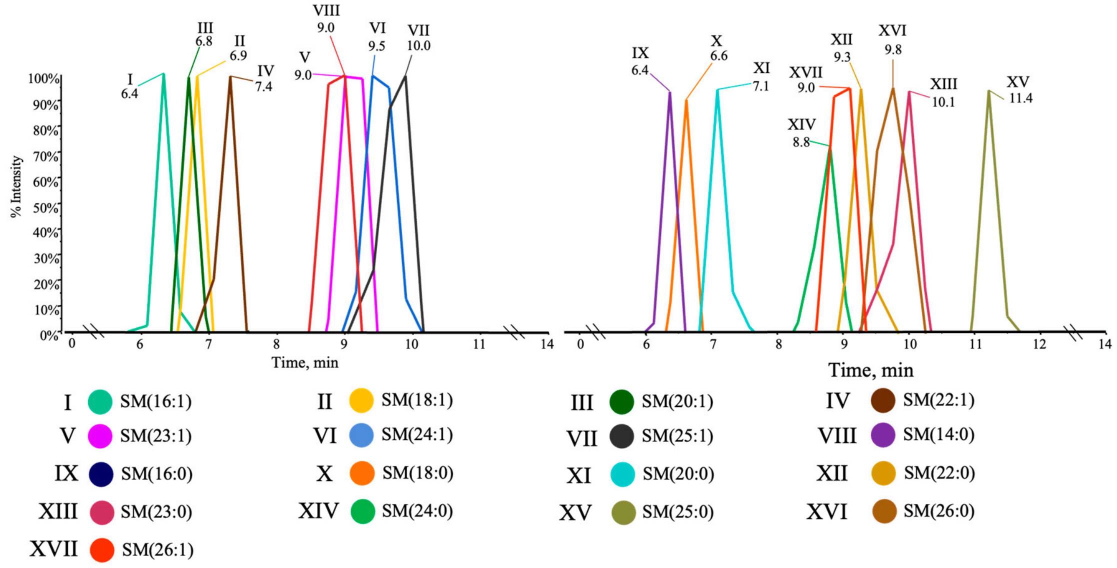

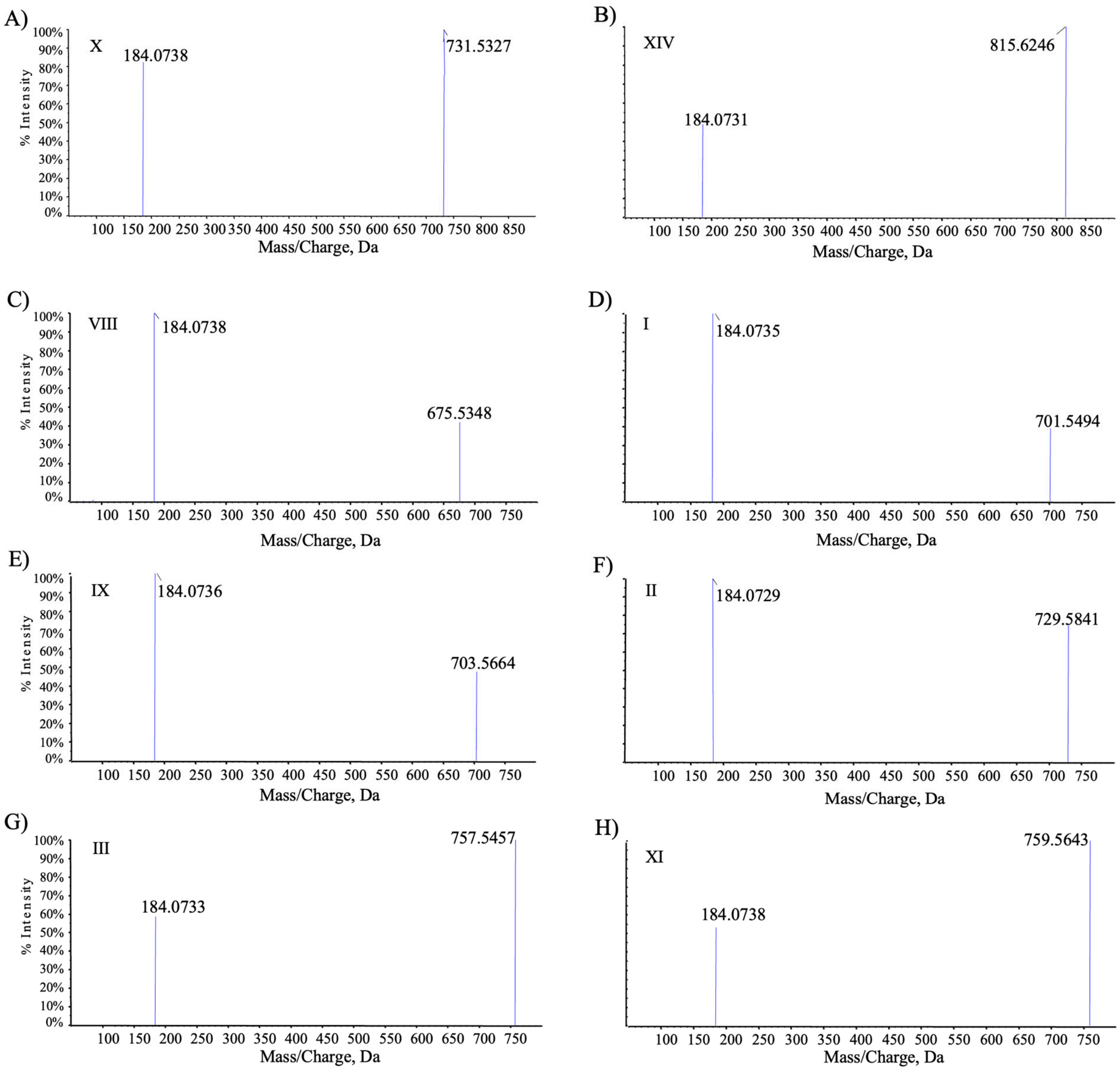

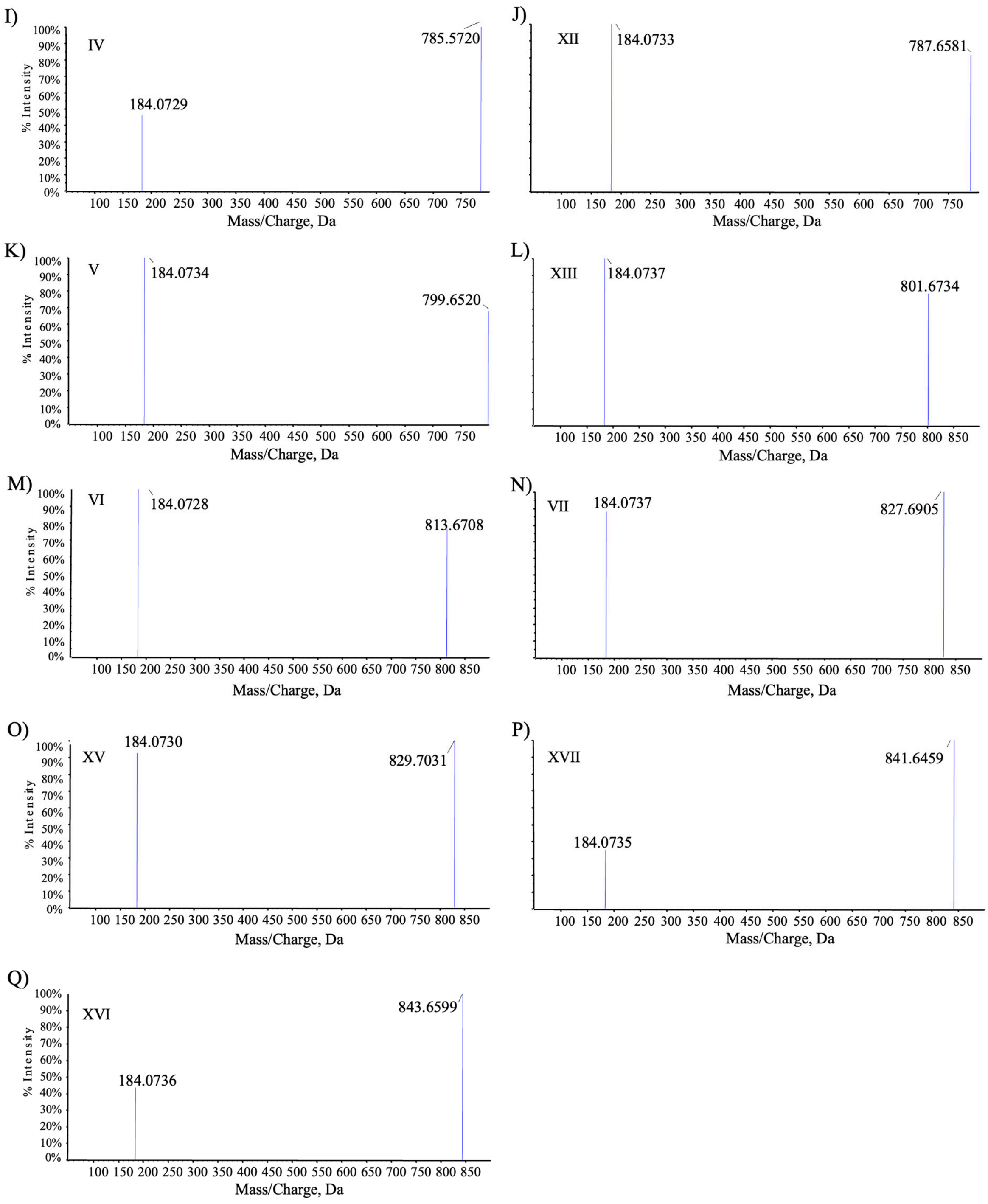

Supplementary Figure S8. Chromatographic profile and mass spectral data of detected sphingomyelins. (I) Sphingomyelin (16:1). (II) Sphingomyelin (18:1). (III) Sphingomyelin (20:1). (IV) Sphingomyelin (22:1). (V) Sphingomyelin (23:1). (VI) Sphingomyelin (24:1). (VII) Sphingomyelin (25:1). (VIII) Sphingomyelin (14:0). (IX) Sphingomyelin (16:0). (X) Sphingomyelin (18:0). (XI) Sphingomyelin (20:0). (XII) Sphingomyelin (22:0). (XIII) Sphingomyelin (23:0). (XIV) Sphingomyelin (24:0). (XV) Sphingomyelin (25:0). (XVI) Sphingomyelin (26:0). (XVII) Sphingomyelin (26:1).

Hexosylceramides - Cer(12:0) IS

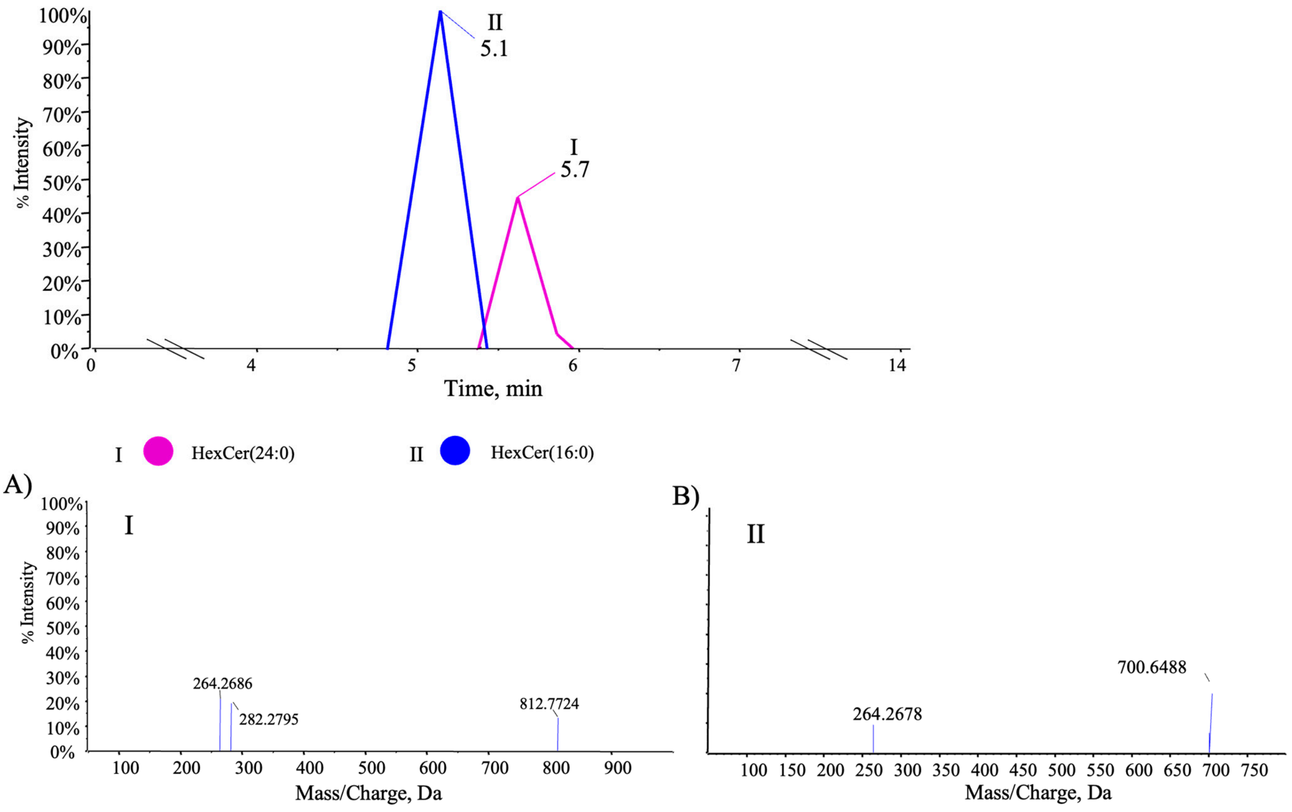

Supplementary Figure S9. Chromatographic profile and mass spectral data of detected hexosylceramides. (I) HexosylCeramide (24:0). (II) HexosylCeramide (16:0).

LacCeramides - LacCer(12:0) IS

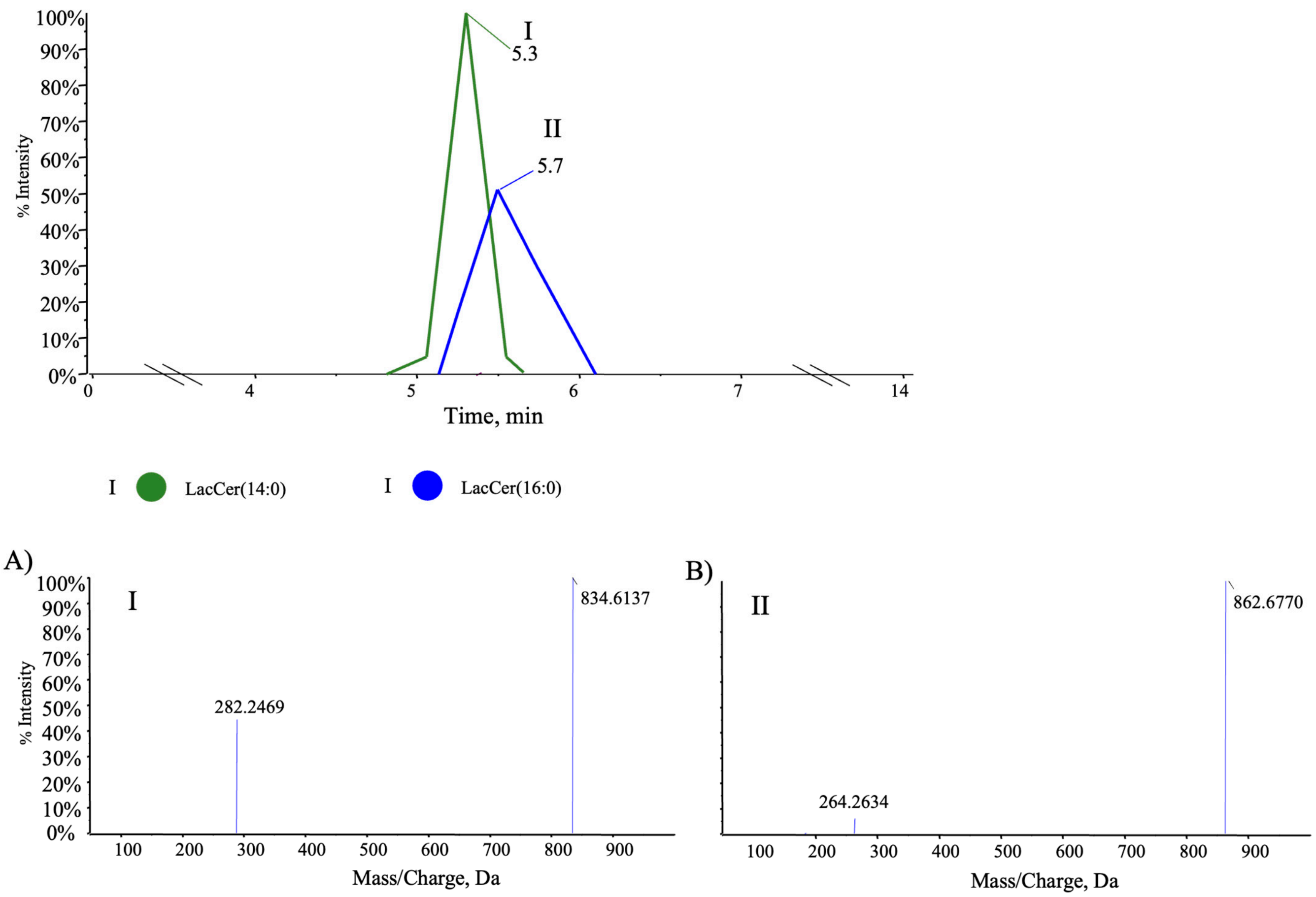

Supplementary Figure S10. Chromatographic profile and mass spectral data of detected lactosylceramides. (I) LacCer (14:0). (II) LacCer (16:0).

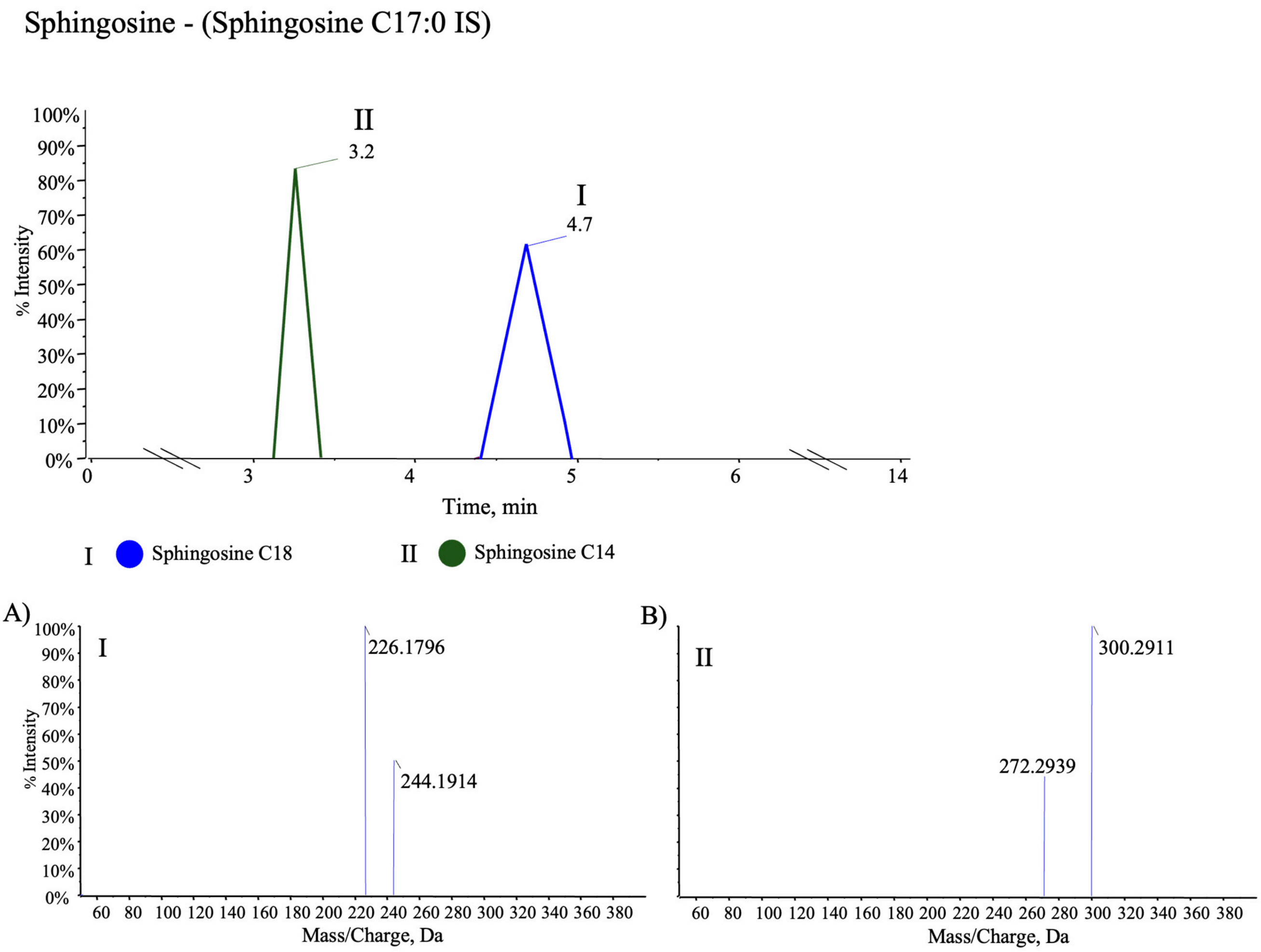

Supplementary Figure S11. Chromatographic profile and mass spectral data of detected sphingosines. (I) Sphingosine (18:0). (II) Sphingosine (14:0).

Sphinganine – (Sphingosine C17:0 IS)

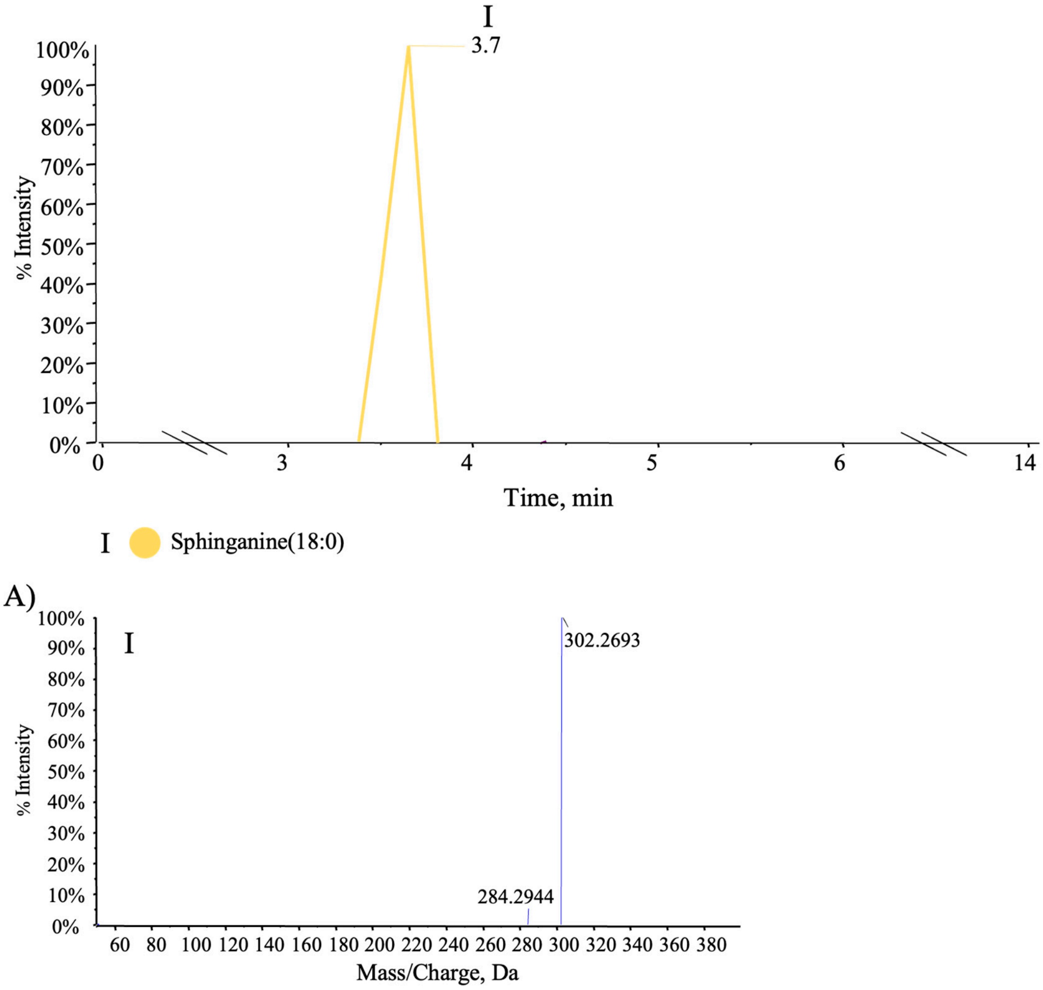

Supplementary Figure S12. Chromatographic profile and mass spectral data of detected sphinganines. (I) Sphinganine (18:0).

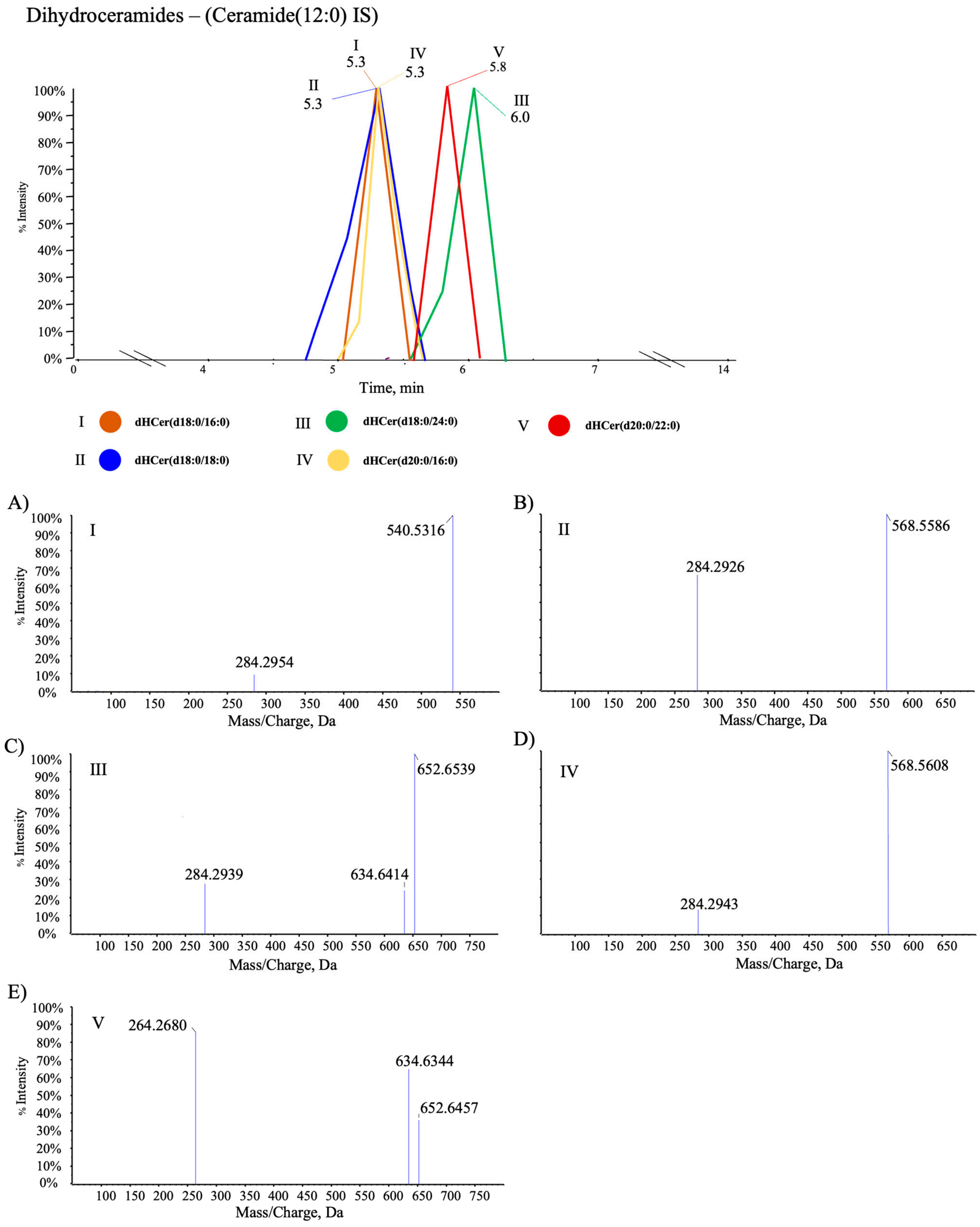

Supplementary Figure S13. Chromatographic profile and mass spectral data of detected dihydroceramides. (I) dHCer(d18:0/16:0). (II) dHCer(d18:0/18:0). (III) dHCer(d18:0/24:0). (IV) dHCer(d20:0/16:0). (V) dHCer(d20:0/22:0).

Supplementary Table S3. List of sphingolipid pathway enzymes and their respective acronyms and ID number.

**Enzyme name**	**Acronym enzyme**	**Acronym Gene**	**HGNC ID**	**NCBI ID**
Serine palmitoyltransferase long chain base subunit 1	SPT	SPTLC1	11277	10558
Serine palmitoyltransferase long chain base subunit 2	SPT	SPTLC2	11278	9517
Serine palmitoyltransferase long chain base subunit 3	SPT	SPTLC3	16253	55304
3-ketodihydrosphingosine reductase	KDR	KDSR	4021	2531
Ceramide synthase 1	CerS	CERS1	14253	10715
Ceramide synthase 2	CerS	CERS2	14076	29956
Ceramide synthase 3	CerS	CERS3	23752	204219
Ceramide synthase 4	CerS	CERS4	23747	79603
Ceramide synthase 5	CerS	CERS5	23749	91012
Ceramide synthase 6	CerS	CERS6	23826	253782
Sphingomyelin synthase 1	SMS	SGMS1	29799	259230
Sphingomyelin synthase 2	SMS	SGMS2	28395	166929
Sphingomyelin phosphodiesterase 1	SMase	SMPD1	11120	6609
Sphingomyelin phosphodiesterase 2	SMase	SMPD2	11121	6610
Sphingomyelin phosphodiesterase 3	SMase	SMPD3	14240	55512
Sphingomyelin phosphodiesterase 4	SMase	SMPD4	32949	55627
N-acylsphingosine amidohydrolase (acid ceramidase)	Csase	ASAH1	735	427
N-acylsphingosine amidohydrolase 2 (Neutral ceramidase/non-lysosomal ceramidase)	Csase	ASAH2	18860	56624
Ceramide synthase 1	CerS	CERS1	14253	10715
Ceramide synthase 2	CerS	CERS2	14076	29956
Ceramide synthase 3	CerS	CERS3	23752	204219
Ceramide synthase 4	CerS	CERS4	23747	79603
Ceramide synthase 5	CerS	CERS5	23749	91012
Ceramide synthase 6	CerS	CERS6	23826	253782
